# Global research trends of nanomaterials application in periodontitis and peri-implantitis: A bibliometric analysis

**DOI:** 10.1016/j.heliyon.2024.e36187

**Published:** 2024-08-13

**Authors:** Chongqing Yu, Dan Zheng, Chi Xu, Tao Wang, Jie Xu

**Affiliations:** a Stomatological Hospital of Chongqing Medical University, No. 426 Songshi North Road, Chongqing, China; b Chongqing Key Laboratory for Oral Diseases and Biomedical Sciences, Chongqing, China; c Chongqing Municipal Key Laboratory of Oral Biomedical Engineering of Higher Education, Chongqing, China

**Keywords:** Nanomaterials, Periodontitis, peri-Implantitis, Bibliometric, Research trend

## Abstract

**Background:**

The application of nanomaterials (NMs) in the treatment of periodontitis and peri-implantitis has shown multifunctional benefits, such as antibacterial properties, immune regulation, and promotion of osteogenesis. However, a comprehensive bibliometric analysis to evaluate global scientific production in this field has not yet been conducted.

**Method:**

We searched for publications related to nanomaterials in periodontitis and peri-implantitis using the WOSCC database. The contributions from institutions, journals, countries, and authors were assessed using VOSviewer, the bibliometrix R package, and Microsoft Excel 2019.

**Results:**

We identified 2275 publications from 66 countries/regions focusing on nanomaterials in periodontitis and peri-implantitis, published between 1993 and 2023. China and the USA were the top contributors in this field, with 653 and 221 publications, respectively. Key topics include antibacterial properties, delivery systems, nanoparticles, and regeneration. The research focus has evolved from traditional treatments to advanced applications of multifunctional nanomaterials.

**Conclusion:**

Significant progress has been made in the application of NMs in periodontitis and peri-implantitis from 1993 to 2023. Future research hotspots will likely focus on multifunctional nanomaterials and those adhering to good manufacturing practices (GMP).

## 1 Introduction

Periodontitis is a condition marked by microbial biofilms associated with teeth, causing chronic inflammation and the deterioration of supporting periodontal tissues. In certain cases, the progression of the disease can result in tooth loss. A comparable situation can arise around dental implants, manifesting as peri-implantitis [1]. Oral microbiota is the etiological unit in periodontitis [2]. As a chronic inflammatory disease, periodontitis is linked with some chronic disorders(such as cardiovascular disease, type 2 diabetes mellitus (T2DM), and Alzheimer's disease) [[3], [4], [5], [6], [7]]. Peri-implantitis, characterized by inflammation in the peri-implant mucosa and the progressive loss of supporting bone, is also caused by bacterial biofilms [[8], [9], [10], [11]]. In 2019, there were 1.1 billion prevalent cases of severe periodontitis globally, which poses a great threat to the world and society [12,13].

At present, treatment approaches for both periodontitis and peri-implantitis involve a range of strategies. Traditional treatments for periodontitis typically include periodontal nonsurgical therapy, which encompasses procedures like supragingival scaling, subgingival scaling, and drug therapy. Surgical interventions for periodontitis, such as bone grafting and guided tissue regeneration, may sometimes be employed. Additionally, repair treatments and periodontal supportive treatments are essential components of comprehensive periodontitis management [[14], [15], [16], [17]]. However, these conventional procedures may lack multifunctionality, such as antibacterial properties, support for periodontal or bone regeneration, and regulation of the immune microenvironment. Therefore, there is an urgent need to develop new approaches to address the complex conditions of periodontitis. Additionally, the most critical aspect of preventing and treating peri-implantitis is implementing anti-infective measures by both patients and dental professionals. Supportive peri-implant care is also necessary for all patients who have undergone implant therapy [18].

In recent years, nanobiotechnology has shown significant potential and value in the medical field, offering new avenues for innovation in dental health [[19], [20], [21]]. Nanomaterials (NMs) and nanomaterial-based treatments are increasingly being utilized in oral health, especially for addressing periodontitis [[22], [23], [24]]. Various nanomaterials are used in oral health, including injectable hydrogels that serve as carriers for drugs, exosomes, and cytokines. Additionally, hydroxyapatite nanoparticles offer benefits such as promoting osteogenesis, exhibiting antibacterial effects, and regulating the local microenvironment. These applications highlight the diverse and promising uses of nanomaterials in the field [[25], [26], [27]].

The exploration of nanomaterials (NMs) in relation to periodontitis and peri-implantitis has been the focus of several studies. However, conducting a comprehensive assessment of research in this interdisciplinary field can be challenging. In recent years, scientometrics—a field that integrates mathematical statistics, computer science, and informatics—has gained prominence as a valuable tool for analyzing and understanding trends in biomedical research [[28], [29], [30]]. Leveraging the tools of mathematics, computer science, bioinformatics, and statistics, bibliometrics facilitates both quantitative and qualitative analyses of research output. This study is the first bibliometric analysis of nanomaterials (NMs) in periodontitis and peri-implantitis. Its primary objectives are to characterize current features, uncover evolutionary trends and connections, identify significant research domains at the intersection of nanomaterials and periodontal health, and provide insights into the future trajectory of this field. By employing bibliometric methods, the study offers a systematic and data-driven approach to understanding the landscape and dynamics of research in this specialized area.

## 2 Material and methods

The search strategy, inclusion and exclusion criteria, data collection, and quality assessment were carefully performed according to PRISMA guidelines [31].

### 2.1 Data resources and search strategy

**Fig. 1:**
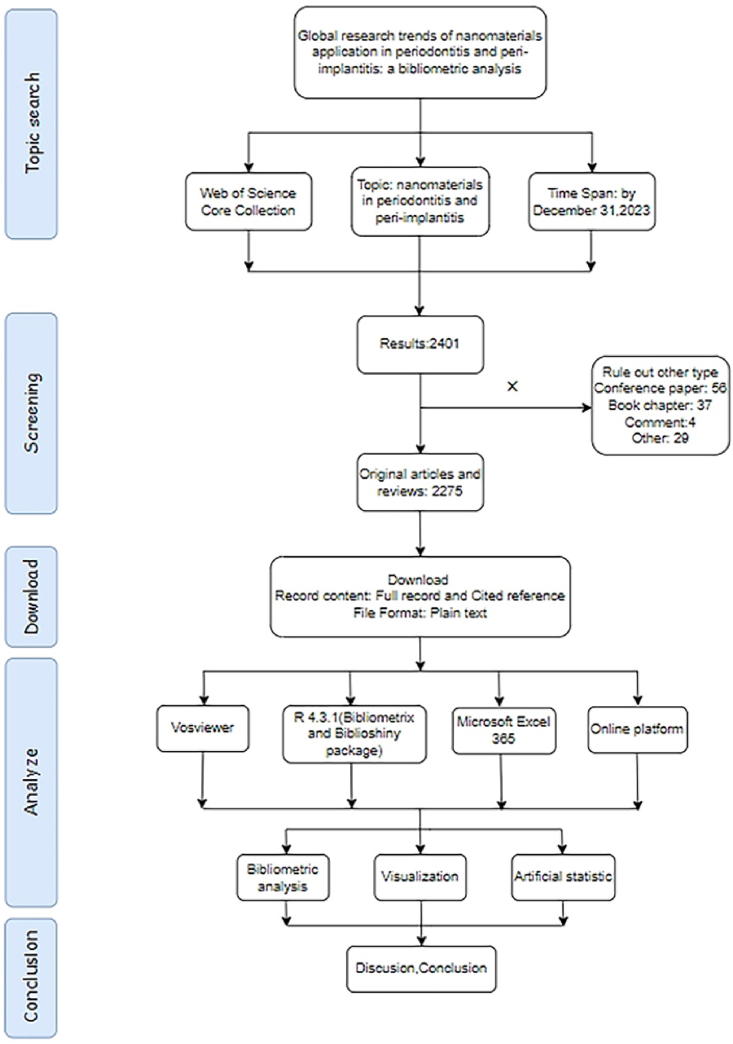
Flow diagram of the study collection process.

Web of Science Core Collection (WOSCC) was the ideal data collection for bibliometric analysis, with more scientific publications than Scopus, CNKI, CSSCI, and other broad databases [32]. All data related to nanomaterials in periodontitis and peri-implantitis was searched in WOSCC. The detailed search strategy was based on previous studies [33,34]. The search strategy and term were as follows: TS=(nano* OR nanoparticle* OR nanodot* OR nanorod* OR nanomaterial* OR nanosphere* OR nanofiber* OR nanotube* OR nanosheet* OR nanotech* OR nanocomposite* ORnanodevice* OR nanowire* OR nanogels* OR nanoliposome* OR nanocarrier* OR nanocluster* OR nanoemulsion* OR nanocrystal* OR nanoconjugate* OR nanodiamod*) AND TS=(peri-implantitis OR Peri Implantitis OR Peri-Implantitides OR Periimplantitis OR Periimplantitides OR peri-implant mucositis OR periodontitis OR periodontal disease OR periodontitis OR periodontal disease OR periodon*). A comprehensive literature review of nanomaterials (NMs) in relation to periodontitis and peri-implantitis was conducted on January 20, 2024. The search was restricted to articles and reviews published up to December 31, 2023, and filtered to include only peer-reviewed English documents from the Web of Science (WOS). The selection process was meticulously managed by a single experienced reviewer following standardized procedures and documenting all relevant parameters. Subsequently, two additional reviewers independently verified the selection process. No automated or computer-aided tools were used. Ultimately, a total of 2275 articles were selected(Fig. 1).

### 2.2 Data analysis

Following a detailed retrieval process, substantial general information on publications was gathered. During the data collection phase, one author led the data collection, while two other authors cross-verified the data to minimize bias and errors. The collected data were then analyzed and visualized using various tools, including Microsoft Excel 365, GraphPad Prism 10.0, VOSviewer 1.6.20, and an online bibliometric platform [35], and R 4.3.2.

## 3 Results

### 3.1 General information and annual publication

The general information and characteristics of publications included in this study are illustrated in Table 1. By 2024, the field comprised 2275 articles from 653 journals, with a total of 94,807 references and 10,018 authors. The annual growth rate of publication number was 18.97 %. The average number of citations per article was 25.37. Additionally, the rate of International co-authorship was 28.17 % in this study.

**Table 1:** General information and characteristics in the publications.

Description	Results
Timespan	1993:2023
Sources (Journals, Books, etc)	653
Documents	2275
Annual Growth Rate %	18.97
Document Average Age	5.47
Average citations per doc	25.37
References	94807
Keywords Plus (ID)	4491
Author's Keywords (DE)	4665
Authors	10018
Authors of single-authored docs	26
Single-authored docs	27
Co-Authors per Doc	6.47
International co-authorships %	28.17

**Fig. 2:**
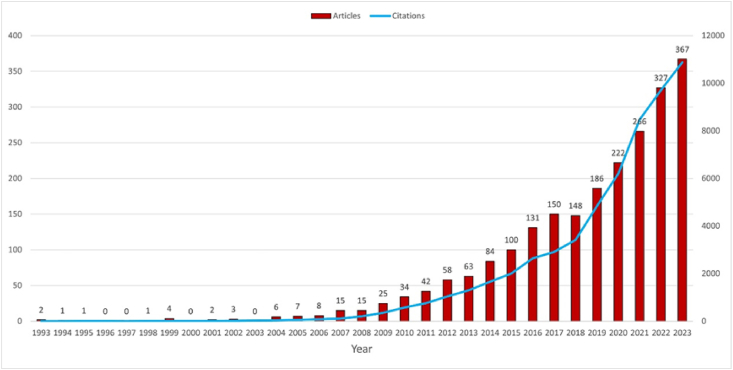
The number of annual publications and citations in the field of NMs in periodontitis and peri-implantitis from 1993 to 2023.

Based on our detailed strategy, a total of 2275 articles related to NMs in periodontitis and peri-implantitis from 1993 to 2023 were included in the present study. The annual publication and citation numbers in this field are shown in Fig. 2. In 2006, only 8 articles were published, reflecting a lack of awareness among scholars about the significance of NMs in periodontitis and peri-implantitis at that time. From 2009 to 2014, the number of publications increased steadily, while it roared dramatically from 2015 to 2023, indicating growing recognition of the potential and value of NMs. The total citation count of NMs in periodontitis and peri-implantitis rose sharply from 2018 to 2023, peaking at 10,878 citations in 2023.

### 3.2 Analysis of countries

**Fig. 3:**
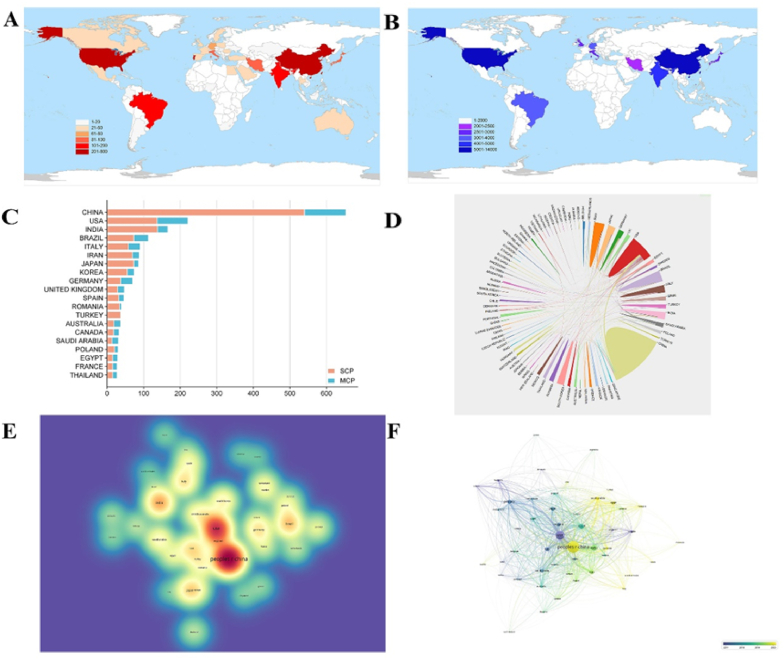
Countries-specific analyses of publications on nanomaterials in periodontitis and peri-implantitis. (A) Distribution map of the number of total publications around the world in the field of NMs in periodontitis and peri-implantitis; (B) Distribution map of number of total citations around the world in the field of NMs in periodontitis and peri-implantitis; (C) The top 10 countries in output(corresponding authors included); (D) International cooperation among countries in the field. In the network visualization, colors are used to represent the countries, and lines indicate international cooperation. The thickness of the line reflects the level of cooperation, with thicker lines indicating closer cooperation; (E)The density visualization of countries' co-citation network.

Analyzing leading countries and regions can provide insights into the development of this field. Therefore, we examined the top 10 most prolific countries and regions in the study of nanomaterials in periodontitis and peri-implantitis (Table 2). Publications for this study originated from 66 countries. Fig. 3A illustrates the distribution of publications in this field, with China and the USA having played predominant roles over the past several decades, ranking as the top two contributors globally. To assess the influence of these publications, we also analyzed total citations by country. China and the USA again ranked as the top two, each with over 10,000 citations (Fig. 3B). Fig. 3C distinguishes between “Multiple Country Publications” (MCP) and “Single Country Publications” (SCP). MCP represents articles with authors from different countries, while SCP denotes articles with authors from the same country. The proportion of MCP provides insights into the level of international collaboration and academic exchange in this field. It is evident that most scientific research exchanges occurred predominantly within individual countries. However, there is a foundation for international cooperation, as MCP constitutes approximately one-third of the publications from the USA.

**Table 2:** Top 10 most prolific countries in the field of NMs in periodontitis and peri-implantitis.

Rank	Country	Total publications	Total citations	Average citation per article	H-index
1	CHINA	653	15281	22.54	59
2	USA	221	13394	34.17	61
3	INDIA	166	4790	25.34	36
4	BRAZIL	113	3700	24.03	33
5	ITALY	90	2927	25.23	31
6	IRAN	88	2150	19.55	26
7	JAPAN	86	2941	24.71	29
8	KOREA	74	2489	25.41	25
9	GERMANY	70	3311	29.56	29
10	UNITED KINGDOM	47	2777	29.54	31

As depicted in Fig. 3D, the international collaboration map delineates China's cooperation with the USA, Australia, and Germany. We could also notice that the USA fostered particularly close collaborations with China, Iran, and Japan among countries/regions. Fig. 3E visualizes publication density among countries, highlighting that China and the USA have significantly higher publication volumes compared to other nations. Fig. 3F illustrates the timeline distribution of contributions from different countries, with the USA being the earliest contributor and China emerging later in the field of nanomaterials in periodontitis and peri-implantitis.

### 3.3 Analysis of institutions

Leading institutions can significantly shape the direction of future research. Thus, we conducted a comprehensive analysis of the top 10 most productive institutions in this field (Table 3). Sichuan University emerged as the leading institution, publishing the highest number of articles on nanomaterials (NMs) in periodontitis and peri-implantitis, highlighting its substantial contribution. Notably, Jilin University achieved the highest total citations, reflecting its significant global impact. Additionally, some institutions, despite having a relatively lower publication count, garnered high average citations per article: the University of California system (33.69), the Chinese Academy of Sciences (33.11), and the University of Michigan (39.24). Sichuan University also led in the h-index for this field, with a value of 23, followed by Jilin University, Universidade Estadual Paulista, and the University of California system.

**Table 3:** The top 10 most prolific institutions in the field of NMs in periodontitis and peri-implantitis.

Rank	Institution name	Number of total publication	Number of total citation	Average citation	H-index
1	Sichuan University	73	1581	21.66	23
2	Egyptian Knowledge Bank(EKB)	53	674	12.72	14
3	Jilin University	51	1583	31.04	22
4	Universidade Estadual Paulista	50	1274	25.48	20
5	Wuhan University	45	1178	26.18	17
6	Nanjing University	44	765	17.39	15
7	University of California system	39	1314	33.69	20
8	Chinese Acaademy of Sciences	37	1225	33.11	18
9	Nanjing Medical University	34	621	18.26	13
10	Shanghai Jiaotong University	33	939	28.45	17
10	University of Michigan	33	1295	39.24	18

**Fig. 4:**
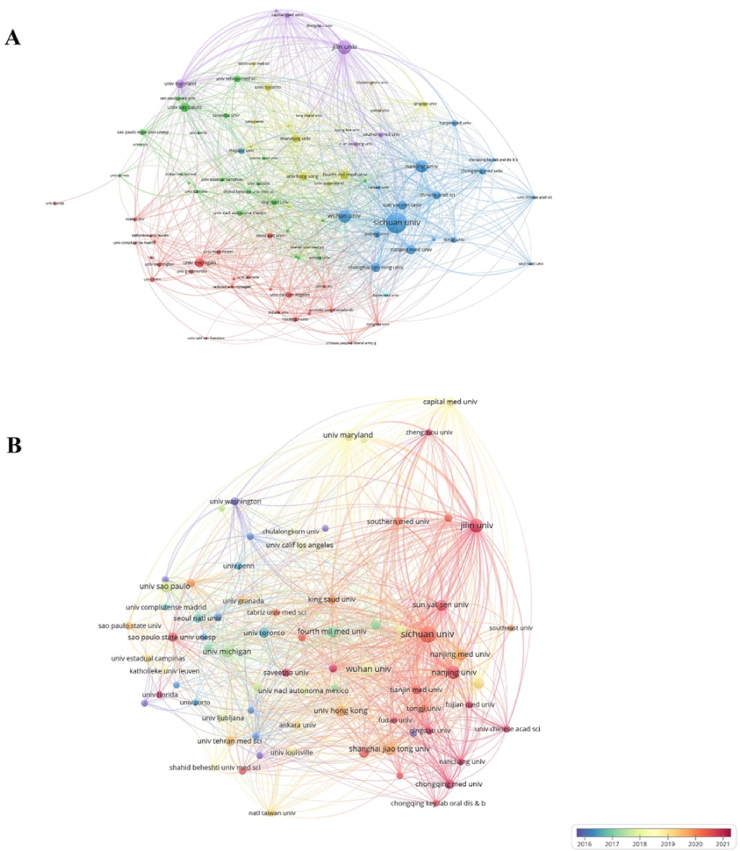
Visualization of institutions analysis (A) Cooperation relationship chart of various institutions. The node size denotes the publication number, and the line thickness illustrates the collaboration strength. (B) The timestamp visualization of institutions' co-occurrence network.

To gain a deeper understanding of collaborations between institutions, we analyzed co-occurrence frequency (Fig. 4A and B). Fig. 4A shows that Sichuan University has formed extensive collaborations with both domestic and international institutions. Similarly, Wuhan University, Nanjing University, and Jilin University have engaged in broad collaborations with various academic entities, highlighting the importance of cooperation in advancing research in this field. Fig. 4B provides insights into institutional cooperation over the past decade, indicating that institutions in the USA (e.g., Washington University, University of Toronto) initiated widespread collaborations earlier. In contrast, Chinese institutions began collaborating later, potentially due to language barriers and limited funding, which may have impeded the development of this field in China.

### 3.4 Analysis of authors

According to the WOSCC, a total of 10,043 authors have contributed to this field. Table 4 provides basic information about the top 10 most prolific authors in NMs research in periodontitis and peri-implantitis. Wang Lin was the most productive author with 27 articles and the highest h-index(19). On the other hand, Bottino Marco received the highest number of citations (1,508) in this field. Moreover, Schwarz, Frank ranked first in average citations per article, indicating that his publications were of significant potential and value.

**Table 4:** The top 10 most prolific authors in the field of NMs in periodontitis and peri-implantitis.

Rank	Author name	Number of publications	Number of total citations	Average citation	h-index
1	Wang,lin	27	1004	37.19	19
2	Bottino, Marco	25	1508	60.32	16
3	Weir, Michael D.	20	894	44.7	16
4	Zhou, Yanmin	18	717	39.83	13
5	Schwarz, Frank	17	1050	61.76	12
6	Qi,Manli	16	559	34.94	10
7	Miao, Leiying	15	229	15.27	7
8	Xu,Hockin H.K.	15	842	56.13	12
9	Sun,Xiao-Lin	13	655	50.38	10
10	Dong, biao	13	553	42.54	10

**Fig. 5:**
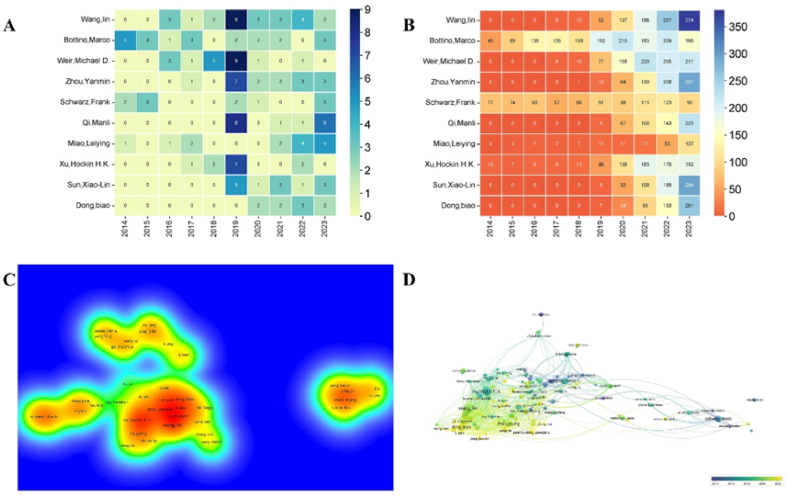
Visualization of authors' analysis. (A) The annual number of publications in the top 10 most prolific authors from 2014 to 2023; (B) Annual number of citations in the top 10 most prolific authors from 2014 to 2023; (C) Density visualization of authors co-cited network; (D) The timestamp visualization of authors co-occurrence network.

To gain a comprehensive understanding of the top 10 most prolific authors, we conducted a detailed analysis (Fig. 5A-D). Fig. 5A illustrates the annual publications of the top 10 most productive authors. Some authors did not contribute any articles in certain years from 2013 to 2023. Notably, in 2019, Wang Lin and Weir Michael D each published 9 articles, marking a particularly productive year for them. Fig. 5B analyzes the annual citations for each author among the top 10. Over the past three years, Wang Lin and Weir Michael D consistently ranked in the top 3 for annual citations, underscoring their significant contributions to the field of NMs in periodontitis and peri-implantitis. Interestingly, despite Weir Michael D publishing only one article from 2021 to 2023 (Fig. 5A), his annual citations remained among the top 2 during these years (Fig. 5B), indicating the lasting impact of his earlier work.

**Fig. 6:**
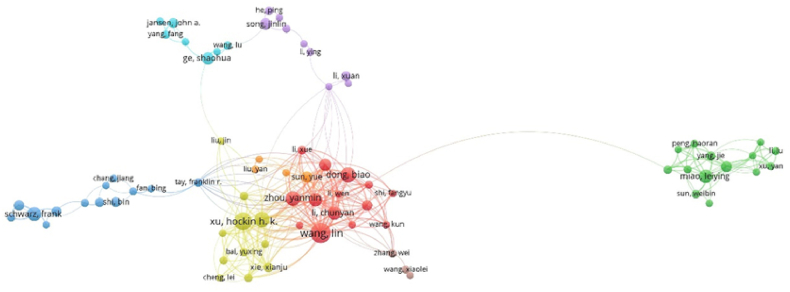
Author collaboration network analysis. Minimum number of documents of each author≥10. The size of nodes suggested the number of articles published by the author.

We conducted a comprehensive density visualization analysis (Fig. 5C). Fig. 5C reveals that Wang Lin has forged extensive collaborations with domestic authors such as Li Chunyan, Li Wen, and Zhou Yanmin, among others. Similar collaboration trends can also be observed among other authors in this field. Fig. 5D offers insights into authors' cooperation over the past decade. It indicates that Wang Lin began collaborating with other scholars around 2020, while Schwarz Frank started engaging in wide collaborations earlier, around 2015. To understand the collaborations between different authors, we employed VOSviewer to create author collaboration network map in Fig. 6. The results illustrated that Wang Lin has collaborated most with other scholars compared with other researchers in this field.

### 3.5 Analysis of journals

**Fig. 7:**
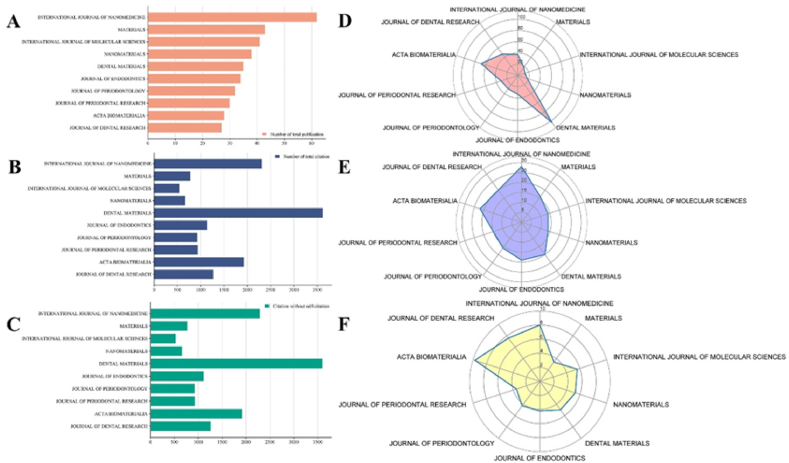
Visualization of journals analysis. (A) A bar chart was used to exhibit the total number of publications and rank of the top ten most productive journals. (B) The total citation number of each journal was obtained from publication to 2023. (C) The total citation number of each journal was obtained from publication to 2023(without self-citation). (D) The average citation of each journal is based on the total publications and citations of one journal. (E) The h-index of the top 10 most productive journals in this field. (F) The Impact factor(IF) of the top 10 most productive journals in this field in 2022.

The significance of identifying high-quality journals and publishing articles in them to reach a broader readership cannot be overstated. Between 1993 and 2023, a total of 657 journals contributed to 2275 articles and reviews in the field of NMs in periodontitis and peri-implantitis. The top 10 most prolific journals published 406 publications, constituting nearly 16 % of all 2275 publications (Table 5). After conducting a thorough analysis, we found that the *International Journal of Nanomedicine* ranked first in the number of total publications and h-index, Dental Materials ranked first in total citations, average citations, and citations without self-citations, while Acta Biomaterialia secured the top position in the 2022 Impact Factor (IF) (Fig. 7A–F, Table 5).

**Table 5:** The top 10 most prolific journals in the field of NMs in periodontitis and peri-implantitis.

Rank	Journal name	Number of total publications	Number of total citation	Average citation
1	INTERNATIONAL JOURNAL OF NANOMEDICINE	62	2315	37.34
2	MATERIALS	43	780	18.14
3	INTERNATIONAL JOURNAL OF MOLECULAR SCIENCES	41	543	13.24
4	NANOMATERIALS	38	670	17.63
5	DENTAL MATERIALS	35	3622	103.49
6	JOURNAL OF ENDODONTICS	34	1140	33.53
7	JOURNAL OF PERIODONTOLOGY	32	930	29.06
8	JOURNAL OF PERIODONTAL RESEARCH	30	940	31.33
9	ACTA BIOMATERIALIA	28	1927	68.82
10	JOURNAL OF DENTAL RESEARCH	27	1270	47.04

**Fig. 8:**
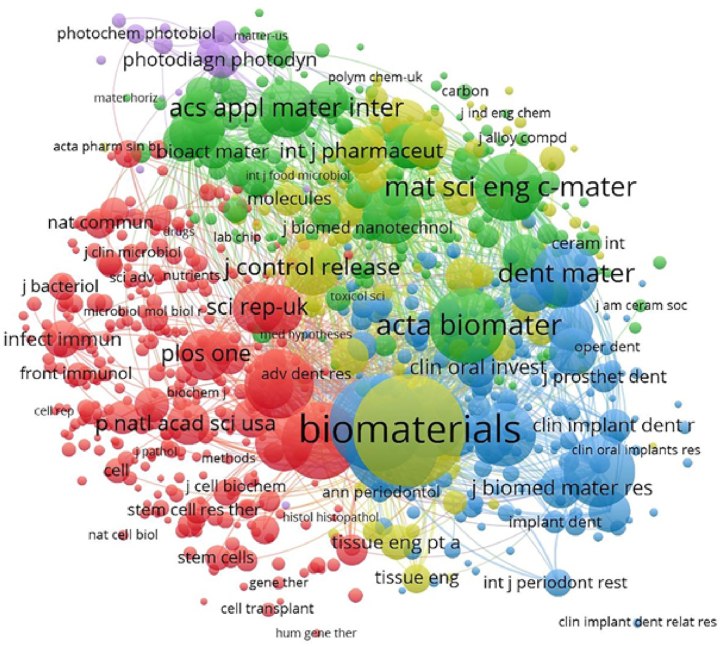
Co-citation analysis between different journals The co-citation analysis between different journals was also conducted using VOSviewer, the journal with a minimal citation of 10 times was limited. As illustrated in Fig. 8, a total of 886 journals were included in the total link strength analysis. The top 3 journals with best total link strength were as follows: Biomaterials(total link strength = 414,072), Journal of Periodontology(total link strength = 244,051), and Journal of Clinical Periodontology(total link strength = 214,565).

In Fig. 7A, a bar chart displays the number of publications for the top ten most productive journals: *International Journal of Nanomedicine* (62) > *Materials* (43) > *International Journal of Molecular Sciences* (41) > *Nanomaterials* (38) > *Dental Materials* (35) > *Journal of Endodontics* (34) > *Journal of Periodontology* (32) > *Journal of Periodontal Research* (30) > *Acta Biomaterialia* (28) > *Journal of Dental Research* (27). Fig. 7B shows the total citation number for each journal: *Dental Materials* (3,622) > *International Journal of Nanomedicine* (2,315) > *Acta Biomaterialia* (1,927) > *Journal of Dental Research* (1,270) > *Journal of Endodontics* (1,140) > *Journal of Periodontal Research* (940) > *Journal of Periodontology* (930) > *Materials* (780) > *Nanomaterials* (670) > *International Journal of Molecular Sciences* (543).

### 3.6 The landmark article and their corresponding author

To identify the landmark publications in the field of NMs in periodontitis and peri-implantitis, we listed the top 10 articles with the most citations in Table 6. As is shown in Table 6, 40 % of the corresponding authors of landmark articles were from China, consistent with the previous analysis of countries, underscoring China's predominant role in this field. The USA, Australia, Germany, India, and South Korea accounted for the other 60 % corresponding authors, highlighting their significant contributions. The publication titled “The Antibacterial Mechanism of Silver Nanoparticles and Its Application in Dentistry” had the highest citation(666 times). This article, authored by Chu CH from China, represents a pivotal work in this domain (see Table 6).

**Table 6:** The top 10 landmark articles in the field of NMs in periodontitis and peri-implantitis.

Rank	Article title	Corresponding authors	Journal	Publication year	Total citations	Corresponding author's country
1	The Antibacterial Mechanism of Silver Nanoparticles and Its Application in Dentistry	Chu, CH	International Journal of Nanomedicine	2020	646	China
2	Polydopamine Nanoparticles as Efficient Scavengers for Reactive Oxygen Species in Periodontal Disease	Yang, XR	ACS Nano	2018	350	China
3	Surface characteristics of dental implants: A review	Hüttig, F	Dental Materials	2018	331	Germany
4	The Therapy of Peri-implantitis: A Systematic Review	Mombelli, A	International Journal of Oral&Maxillofacial Surgery	2014	258	Australia
5	General review of titanium toxicity	Kim, SM	International Journal of Implant Dentistry	2019	199	South Korea
6	Electrospun polymeric nanofibers: New horizons in drug delivery	Misra, M	European Journal of Pharmaceutical Sciences	2017	178	India
7	2D MOF Periodontitis Photodynamic Ion Therapy	Wu, SL	Journal of The American Chemical Society	2021	144	China
8	Recent advances in biomedical engineering of nano-hydroxyapatite including dentistry, cancer treatment and bone repair	Song, C	Composites Part B-Engineering	2021	127	China
9	The oralome and its dysbiosis: New insights into oral microbiome-host interactions	Kapila, YL	Computional and Structural Biotechnology Journal	2021	122	USA
10	The Impact of Dental Implant Surface Modifications on Osseointegration and Biofilm Formation	Li, CS	Journal of Clinical Medicine	2021	84	USA

### 3.7 Analysis of keywords

**Fig. 9:**
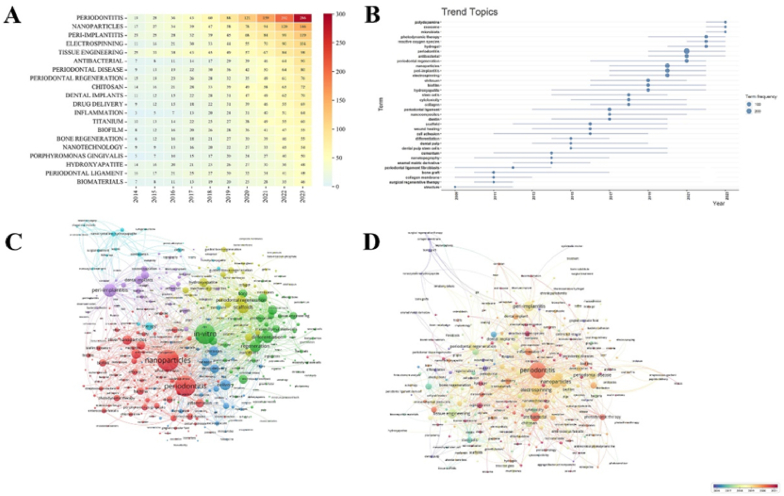
Visualization of keywords analysis. (A) A heatmap was used to show the annual occurrence frequency of the top 10 most common keywords. A rectangle chart with yellow means less frequency of keywords and a rectangle chart with blue means more frequency. (B) Trend topics map of author's keyword for publications published between 1993 and 2023. (C) Network visualization of co-occurrence author keywords. Circle size is based on the number of occurrences. (D) The timestamp visualization of all keywords co-occurrence network.

Keyword analysis plays a crucial role in helping scholars understand trends and hotspots in the field of NMs in periodontitis and peri-implantitis. Table 7 lists the top 30 most common keywords according to Web of Science (WOS). Among these keywords, “In-vitro,” “nanoparticles,” “differentiation,” “delivery,” and “scaffolds” rank as the top 5 most frequently occurring, indicating that much of the research is conducted in vitro. The annual occurrence frequency of the top 10 keywords is illustrated in a matrix heatmap (Fig. 9A). In 2023, only the occurrence frequency of “Periodontitis,” “Nanoparticles,” “Peri-implantitis,” and “Electrospinning” exceeded 100, highlighting them as hotspots in the field. Using VOSviewer for burst detection, we identified the top 35 keywords with the highest burst strength, as shown in Fig. 9B. Notably, “periodontal regeneration” emerged as a hotspot in 2016, while “antibacterial” gained prominence in 2019. Recently, keywords like “polydopamine,” “exosome,” and “microbiota” have also emerged as significant areas of interest, reflecting ongoing scholarly attention in the field.

**Table 7:** The top 30 most frequent keywords in NMs in periodontitis and peri-implantitis.

Keywords	Occurrences	Keywords	Occurrences	Keywords	Occurrences
in-vitro	385	cells	113	hydroxyapatite	83
nanoparticles	285	release	111	disease	82
differentiation	164	silver nanoparticles	108	chitosan	81
delivery	155	bone	103	adhesion	80
scaffolds	152	mesenchymal stem-cells	102	tissue	78
regeneration	133	stem-cells	100	porphyromonas-gingivalis	77
periodontitis	125	therapy	100	controlled-release	74
expression	121	osteogenic differentiation	94	mechanical-properties	74
peri-implantitis	120	proliferation	91	bacteria	73
drug-delivery	119	antibacterial activity	89	bone regeneration	72

Additionally, we conducted a density visualization of keyword co-occurrence (Fig. 9C), providing essential insights into the interconnections between keywords. The majority of current research focuses on experiments related to nanoparticles, highlighting their significance in the treatment of periodontitis and peri-implantitis. Fig. 9D color-codes each node based on the average occurrence time of the keyword. Most high-frequency keywords appeared before 2019, indicating that early research was concentrated in these areas. Keywords such as “antibacterial,” “inflammation,” and “photodynamic therapy,” which emerged after 2019, suggest that recent research hotspots are primarily focused on the role of NMs in periodontitis and peri-implantitis.

**Fig. 10:**
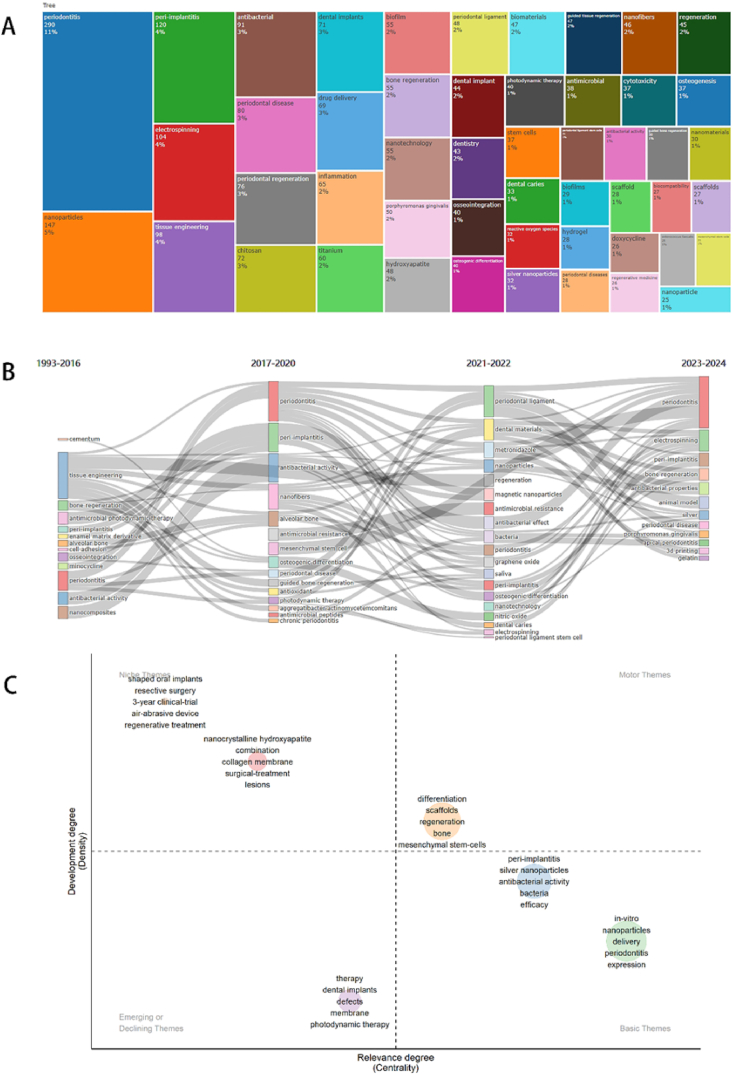
(A) Treemap of the keywords; (B) Thematic evolution alluviation map; (C) Thematic map.

Based on keyword analysis, we can discern foundational trends in the field of NMs in periodontitis and peri-implantitis. However, identifying emerging hotspots remains challenging. To address this, we conducted a treemap analysis using bibliometric tools to evaluate the prevalence of different keywords (Fig. 10A). Fig. 10A reveals that “periodontitis” and “nanoparticles” are the two most prevalent keywords, accounting for 11 % and 5 % of occurrences, respectively. Additionally, a bibliometric analysis of influential keywords over different time periods(Fig. 10B) highlights the evolution of research focus:(1)1993 to 2006: Influential keywords included “tissue engineering,” “periodontitis,” and “antibacterial activity”.(2)2017 to 2020: New influential keywords emerged, such as “peri-implantitis” and “nanofibers.“(3)2021 to 2022: Research attention shifted to “periodontal ligament,” “dental materials,” and “metronidazole.“(4)2023 to 2024: Keywords like “electrospinning” and “bone regeneration” became prominent.

To assist scholars in decision-making, we employed a “Thematic Map” based on a dataset of 4498 author keywords (Fig. 10C). Using the “Walktrap” algorithm with a Random Walk strategy, we conducted a community discovery analysis. The visualization plots keywords along the x-axis, representing their significance in the field, and the y-axis, indicating the level of development of these research signatures. The key points in four distinct thematic quadrants were as follows: Firstly, Motor Themes(First Quadrant), which denote topics that are both important and well-developed. In this quadrant, only one well-established and significant theme emerged. The orange mode theme includes keywords such as “differentiation”, “scaffolds”, “regeneration”, “bone” and “mesenchymal stem cells”. These themes have a relevance degree, indicating their importance in this field, but are not yet central enough. In addition, Niche Themes(Second Quadrant), suggest that the topic has developed well but is not crucial to the current field. This quadrant included two themes. The red mode theme includes keywords such as “nanocrystalline hydroxyapatite”, “combination”, “collagen membrane”, “surgical-treatment” and “lesions”. The brown mode theme includes keywords such as “shaped oral implants”, “respective surgery”, “3-year clinical trial”, “air abrasive device” and “regenerative treatment”. some keywords such as “collagen membrane”, “surgical treatment” and “shaped oral implants”. They are not highly related to the current field. Furthermore, Emerging or Declining Themes (Third Quadrant), stand for marginal themes and may not be well developed or may just emerge or are about to disappear. It includes one theme. The purple mode theme includes keywords such as “photodynamic therapy” and “defect”. Lastly, Basic Themes(Fourth Quadrant), are topics that are critical to the field but have not been well developed. It includes two themes. The blue mode theme includes keywords such as “peri-implantitis”, “silver nanoparticles”, “antibacterial activity”, “bacteria” and “efficacy”. The green mode theme includes keywords such as “in-vitro”, “nanoparticles”, “delivery”, “periodontitis” and “expression”. In short, this thematic map provides a strategic overview of current research trends and emerging areas within the field of NMs in periodontitis and peri-implantitis.

## 4 Discussion

Periodontitis and peri-implantitis are significant oral health issues that impact both natural and artificial teeth. Over the last three decades, numerous studies have investigated the application of nanomaterials in addressing these conditions, recognizing their substantial value. This bibliometric analysis has employed various tools to evaluate the current characteristics and predict future research hotspots in this field.

### 4.1 General information

This study represents, to our knowledge, the first comprehensive unveiling of a global knowledge map detailing NMs involvement in periodontitis and peri-implantitis across the past three decades. Employing scientometric metrics and information visualization techniques, this research encapsulates the temporal and spatial distribution of publications, and their respective contributions, as well as insights into the evolutionary trends and interconnected networks among authors, journals, countries, and institutions in this field. The publication and citation number could serve as important indicators in a given research field. Many publications and citations in the field of NMs in periodontitis and peri-implantitis witnessed a dramatic increase over the past three decades, without evident fluctuations. These results indicate that the NMs in periodontitis and peri-implantitis have raised increasing attention and have been soaring over the past thirty years.

After an in-depth analysis of predominant countries/regions in this field, we found that China and the USA occupied significant status in the field of NMs in periodontitis and peri-implantitis, not only concerning their total publications but also citations. Other researchers also analyzed the NMs in stomatology employing the bibliometric method. These two countries also accounted for the top 2 in this field [36].

Nevertheless, we also found that the publications rank was not consistent with the citations rank in this field, possible reasons might be that several scholars focused on the number of publications not the depth of research. The USA ranked top one in the average article citation and h-index, indicating its contribution to this field. The international collaboration map illustrated that the USA and China cooperated with other countries widely. In short, China has published most articles in this field over the past decades, but Chinese research in this field lacks the influence recognized and evaluated by the world. As for the leading institution in the field of NMs in periodontitis and peri-implantitis, there were seven institutions in China in the top 10 most prolific institutions. Similar to leading countries, we also noticed that institutions in China obtained corresponding citation ranks, suggesting their great global impact. Besides, The distribution of institutions reveals China's dominant position in this field. We advocate for collaboration in this field to transcend geographical and political barriers, encouraging scholars to actively engage in collaborative exchanges with their international counterparts. This approach fosters a more expansive and fruitful exchange of ideas, knowledge, and expertise, ultimately advancing the progress and impact of research in this domain.

Among all the authors in the field of nanomaterials (NMs) in periodontitis and peri-implantitis, we conducted a detailed analysis of the top 10 most prolific authors. Wang Lin emerged as the most productive author with 27 articles, while Bottino Marco secured the highest number of citations (1,508) in this field. Schwarz Frank claimed the top position in the average citation, indicating significant contributions to the field. It's noteworthy that some authors focused on quantity rather than the depth of their articles, resulting in lower citations compared to other scholars. Policymakers could consider initiatives to improve publication quality over quantity, enhancing global impact. Additionally, our analysis of the top 10 most productive journals revealed that the International Journal of Nanomedicine had the highest publication number, while Dental Materials garnered the most citations among these journals.

Keywords in articles serve as pivotal labels through which authors articulate their research topics, methodologies, and crucial viewpoints. These succinct terms play a crucial role in summarizing and highlighting the essence of the research conducted within the articles. The “emerging keywords” refer to the keywords that exhibit a drastic increase in a certain period in frequency of use during a period. Keywords often mirror the forefront issues within a research field. Employing keyword emergence analysis based on keyword co-occurrence enables the visual representation of focal points, hot topics, and the evolving trends within this field. This analysis serves as a valuable tool to discern the most pertinent and burgeoning areas of advancement. Our analysis revealed a persistent emergence of keywords such as “polydopamine,” “exosome,” “microbiota,” and “photodynamic therapy” continuing until 2024. These keywords signify ongoing and sustained relevance, suggesting their continued significance in the field's advancements. In addition, we carried out a thematic map analysis to make clear future research trends. Some keywords related to periodontal regenerative medicine could be seen in Motor themes(i.e. “differentiation”, “scaffolds”, “regeneration”, “bone” and “mesenchymal stem-cells”). Nonetheless, competition is fierce, making significant breakthroughs challenging. Basic themes represent areas that are not yet mature in this field but possess high centrality and low density. They primarily center on the treatment of both periodontitis and peri-implantitis (i.e. “silver nanoparticles”, “antibacterial activity”, “delivery”, “bacteria”, etc.). Both Motor themes and Basic themes have been well explored with advanced techniques associated with NMs and involved much basic research in the treatment of periodontitis and peri-implantitis. Therefore, future development will also be closely related to new treatments or management for these two diseases. Moreover, the thematic map also included “emerging or declining themes”. In this study, this theme mentioned keywords including “defects”, “membrane” and “photodynamic therapy”, indicating that photodynamic therapy-related studies were emerging in the field of NMs in periodontitis and peri-implantitis.

### 4.2 The hotpots and trends

Based on the burst of keywords analysis, we could observe the dynamic alternation in this field. According to Fig. 9B, nanomaterials with various components (such as exosome, and polydopamine) are emerging trends in this field. Besides, microbiota, reactive oxygen species(ROS), photodynamic therapy, and hydrogel are also hotspots in the future trend.

Nanotechnology, with a focus on nano-sized materials such as nanoparticles, scaffolds, and hydrogels, has indeed become a pivotal tool in the treatment of periodontitis and peri-implantitis. These nano-sized materials offer advantages in various aspects, including drug delivery, antibacterial effects, immune regulation, osteogenesis, and reactive oxygen species (ROS) scavenging, which is consistent with the main therapeutic strategies for periodontitis(antibacterial agents, immune regulation) [37]. Besides, novel modification strategies for Ti-implant also included antibacterial, anti-inflammatory, and osteogenecity [38]. This is consistent with the results of this study.

The top 10 landmark publications listed in Table 6 likely provide comprehensive insights into the advancements and key aspects of nanomaterial applications in the field. The prominence of antibacterial functions, as indicated by the top-ranked publication exploring the antibacterial mechanisms of silver nanomaterials, underscores the significance of addressing microbial challenges in dental health. Nanotechnology's ability to precisely manipulate materials at the nanoscale opens up new possibilities for designing targeted and efficient therapies, contributing to the evolving landscape of treatment strategies for periodontitis and peri-implantitis. These landmark publications likely serve as foundational resources for understanding the key principles and mechanisms driving advancements in this field.

Reactive oxygen species (ROS) play a crucial role in the immune response against pathogens in the context of periodontitis. This has been verified in previous studies [39,40], and novel NMs aimed to address this issue. Zhu et al. constructed a biosafe injectable hydrogel drug-controlled delivery system loaded with quercetin to treat oxidative stress injury in periodontal tissues [41]. Additionally, Yingying Xu et al. proposed one microenvironment-responsive metal-phenolic nanozyme release platform scavenging multiple ROS [42]. While ROS are essential for the host defense mechanism, excessive and uncontrolled production of ROS can contribute to tissue damage and exacerbate the progression of periodontal diseases, leading to bone defects [43]. Nanomaterials and photodynamic therapy (PDT) offer innovative approaches to managing the intricate conditions associated with periodontitis. Nanomaterials can be designed to scavenge or modulate ROS levels, helping to maintain a balanced immune response without causing excessive tissue damage. PDT, as mentioned earlier, utilizes photosensitizers activated by light to generate reactive oxygen species selectively, providing a targeted antibacterial effect [44]. The integration of nanomaterials and PDT in the treatment of periodontitis reflects the interdisciplinary efforts to develop precise and effective therapeutic strategies, addressing both the microbial challenge and the host immune response to promote periodontal health. When combined with nanomaterials, PDT can be enhanced in terms of precision, targeting, and delivery of the photosensitizer. Nanomaterials can serve as carriers for photosensitizers, improving their stability, bioavailability, and interaction with target tissues. This combination has shown promising results in the treatment of periodontal diseases by selectively targeting and eliminating pathogenic microorganisms associated with periodontitis [[44], [45], [46]]. Exosomes have gained attention in periodontitis treatment. These small vesicles, secreted by various cell types, play a crucial role in cellular communication and are essential contributors to cell efficacy. Exosomes serve as natural carriers of functional small RNA and proteins, making them valuable for therapeutic interventions in conditions like periodontitis [47]. Indeed, the potential applications of exosomes in the diagnosis and treatment of diseases, including periodontitis, are gaining popularity. Exosomes derived from mesenchymal stem cells (MSCs), whether used alone or in combination with biomaterials, hold significant promise for cell-free treatment and tissue regeneration in the context of periodontitis [[48], [49], [50]].

While the applications of nanomaterials (NMs) in addressing periodontitis and peri-implantitis have shown promise in previous studies, there is still much work to be done to transition these findings into clinical practice. Clinical trials involving NMs must adhere to good manufacturing practice (GMP), and efforts should be directed toward developing more convenient and efficient manufacturing technologies to enhance the homogeneity, purity, and repeatability of NMs. Additionally, research should delve into understanding the specific roles and mechanisms of NMs in the context of periodontitis and peri-implantitis more comprehensively. The interaction mechanisms between NMs and the host remain unclear, posing challenges in accurately regulating target cells and functions. Despite these challenges, NMs hold the potential to offer personalized medical strategies for preventing and treating periodontitis and peri-implantitis.

### 4.3 Limitations and strengths

Several limitations were present in this study. Primarily, the reliance solely on the WOSCC, chosen for its standardized and comprehensive database, posed limitations as other databases, like Embase or Cochrane Library, were not included. This reliance might have resulted in some data omissions. Furthermore, while this study provided a detailed scientometric and visualization analysis in the field of NMs in periodontitis and peri-implantitis, it should be considered as a reference for scholars in this domain. As the field continually evolves, future studies will play a significant role in offering a more comprehensive view of the subject matter.

However, this study serves as a foundational resource for scholars exploring NMs in the treatment of periodontitis and peri-implantitis. Future research endeavors will play a crucial role in overcoming these constraints, offering a more comprehensive understanding of this field. Encouragingly, we anticipate increased scholarly attention toward nanomaterials and their potential mechanisms in periodontitis and peri-implantitis.

## 5 Conclusion

Research on nanomaterials in periodontitis and peri-implantitis has gained significant traction in the research community since the late 20th century. Collaborations among various countries, institutions, and authors in this field have fostered rapid advancements in this field. Both the exploration of fundamental mechanisms and the development of advanced nanomaterials hold promise for enhancing clinical practices in periodontitis and peri-implantitis management. Emphasizing attention on clinical applications, researchers aim to significantly improve medical care for patients with periodontitis or peri-implantitis.

## Funding

This work was supported by the 10.13039/100014717National Natural Science Foundation of China(Grant No. 81800999), Project supported by Chongqing 10.13039/100018696Health Commission & Science and Technology Bureau Foundation(NO.2023ZDXM019)

## Data availability statement

The data in this study can be found here: https://www.webofscience.com/wos/alldb/basic-search.

## CRediT authorship contribution statement

**Chongqing Yu:** Supervision, Resources. **Dan Zheng:** Data curation, Conceptualization. **Chi Xu:** Data curation, Conceptualization. **Tao Wang:** Supervision, Resources. **Jie Xu:** Visualization, Validation, Supervision.

## Declaration of competing interest

The authors declare the following financial interests/personal relationships which may be considered as potential competing interests:

Jie Xu reports financial support was provided by 10.13039/501100001809National Natural Science Foundation of China. Jie Xu reports financial support was provided by Project supported by Chongqing 10.13039/100018696Health Commission & Science and Technology Bureau Foundation. If there are other authors, they declare that they have no known competing financial interests or personal relationships that could have appeared to influence the work reported in this paper.
